# Confirmation of spatial patterns and temperature effects in Bluetongue virus serotype-8 transmission in NW-Europe from the 2007 reported case data

**DOI:** 10.1186/s13567-014-0075-x

**Published:** 2014-08-09

**Authors:** Gert Jan Boender, Thomas J Hagenaars, Armin RW Elbers, Jörn M Gethmann, Estelle Meroc, Helene Guis, Aline A de Koeijer

**Affiliations:** Department of Epidemiology, Crisis management and Diagnostics, Central Veterinary Institute (CVI), part of Wageningen UR, P.O. Box 65, NL-8200 AB Lelystad, Netherlands; Friedrich-Loeffler Institut, Institute of Epidemiology, Wusterhausen, Germany; Veterinary and Agrochemical Research Centre (CODA-CERVA), Brussels, Belgium; CIRAD, UMR CMAEE, Montpellier, France

## Abstract

Two separate analyses were carried out to understand the epidemiology of Bluetongue virus serotype 8 (BTV-8) in 2007 in North West Europe: First, the temporal change in transmission rates was compared to the evolution of temperature during that season. Second, we evaluated the spatio-temporal dynamics of newly reported outbreaks, to estimate a spatial transmission kernel. For both analyses, the approach as used before in analysing the 2006 BTV-8 epidemic had to be adapted in order to take into account the fact that the 2007 epidemic was not a newly arising epidemic, but one advancing from whereto it had already spread in 2006. We found that within the area already affected by the 2006 outbreak, the pattern of newly infected farms in 2007 cannot be explained by between-farm transmission, but rather by local re-emergence of the virus throughout that region. This indicates that persistence through winter was ubiquitous for BTV-8. Just like in 2006, we also found that the temperature at which the infection starts to spread lies close to 15 °C. Finally, we found that the shape of the transmission kernel is in line with the one from the 2006 epidemic. In conclusion, despite the substantial differences between 2006 and 2007 in temperature patterns (2006 featured a heat wave in July, whereas 2007 was more regular) and spatial epidemic extent, both the minimum temperature required for transmission and the transmission kernel were similar to those estimated for the 2006 outbreak, indicating that they are robust properties, suitable for extrapolation to other years and similar regions.

## Introduction

In 2006 BTV-8 invaded North West (NW) Europe. At the time, little was known about the dynamics of this infection. Therefore, the abundance and dynamics of the vector was investigated [1]. To learn more about the quantitative aspects of the infection dynamics, we analysed the BTV-8 transmission dynamics in NW Europe, based on the joint data from the countries affected in the 2006 epidemic [2]. Because the climatic changes suggest increasing risk of BTV infections in Europe [3] and repeated occurrences of various strains support this idea [4,5], the need for good quantitative risk assessments increases [6]. This is particularly relevant for areas where little data has been gathered and analysed in the past [7]. Thus quantitative knowledge regarding BTV transmission and the impact of temperature and spatial aspects on the transmission are important. Here, in order to gain such knowledge, a previous analysis of the 2006 data was followed by an analysis of the data collected during the epidemic in 2007.

Specifically, we analysed the influence of temperature and the scale of spatial spread. In 2007, the infection persisted and spread again during the vector active season. By the end of 2007, most ruminant farms in Belgium and the Netherlands had been infected, while a large part of France and Germany were also infected. During the following years, a vaccination campaign was carried out in the infected areas, after which the infection disappeared from these areas.

In the analysis of the 2006 epidemic it was found that the infection spreads between ruminant herds only when the daily mean temperature exceeds 15 °C, and that the spatial transmission is influenced by the transport restriction regulations of the epidemic [2]. For example, it was found that under transport restrictions using a 20-km zone in 2006, 85% of the transmission between farms was limited to distances within a 20 km range.

The year 2006 was a meteorologically exceptional year featuring an extremely warm summer period [8]. Therefore, it remained a question to what extent the results and conclusions would apply to other weather conditions. To address this question, we analysed the BTV-8 outbreak data from 2007, during which new farms in an extended area were affected under different weather conditions.

## Materials and methods

### Data and data handling

In the framework of the European FP6 Network of Excellence of Diagnostics and Control of Epizootic Diseases EPIZONE [9], National Reference Laboratories from Germany (FLI), the Netherlands (CVI) and Belgium (CODA-CERVA), together with the Centre de Coopération Internationale en Recherche Agronomique pour le Développement (CIRAD), Montpellier, France collaborated on an epidemiological analysis of the BTV-8 epidemic in ruminant herds in 2006 and 2007.

The epidemiological data we used were the following: the geographical coordinates for each outbreak farm (in total the datasets include 40 927 reported farms) and the date when clinical suspicion was reported to veterinary authorities. Furthermore, we used information on the number of all farms housing cattle, sheep and goats per municipality as administrative unit. FLI provided a secure database platform and server making these and some further background data on the 2007 BTV-8 epidemic available to all group members. The number of non-outbreak farms per administrative unit has a mean of 16, a standard deviation of 41, a minimum of 0, a maximum of 1668, while the quartiles are: Q_1_ = 3, Q_2_ = 7, and Q_3_ = 15. The nearest-neighbour distance between administrative units has a mean of 3.1 km, a standard deviation of 1.4 km, a minimum of 0 km, a maximum of 48.6 km, while the quartiles are: Q_1_ = 2.2 km, Q_2_ = 2.8 km, and Q_3_ = 3.7 km.

**Figure 1:**
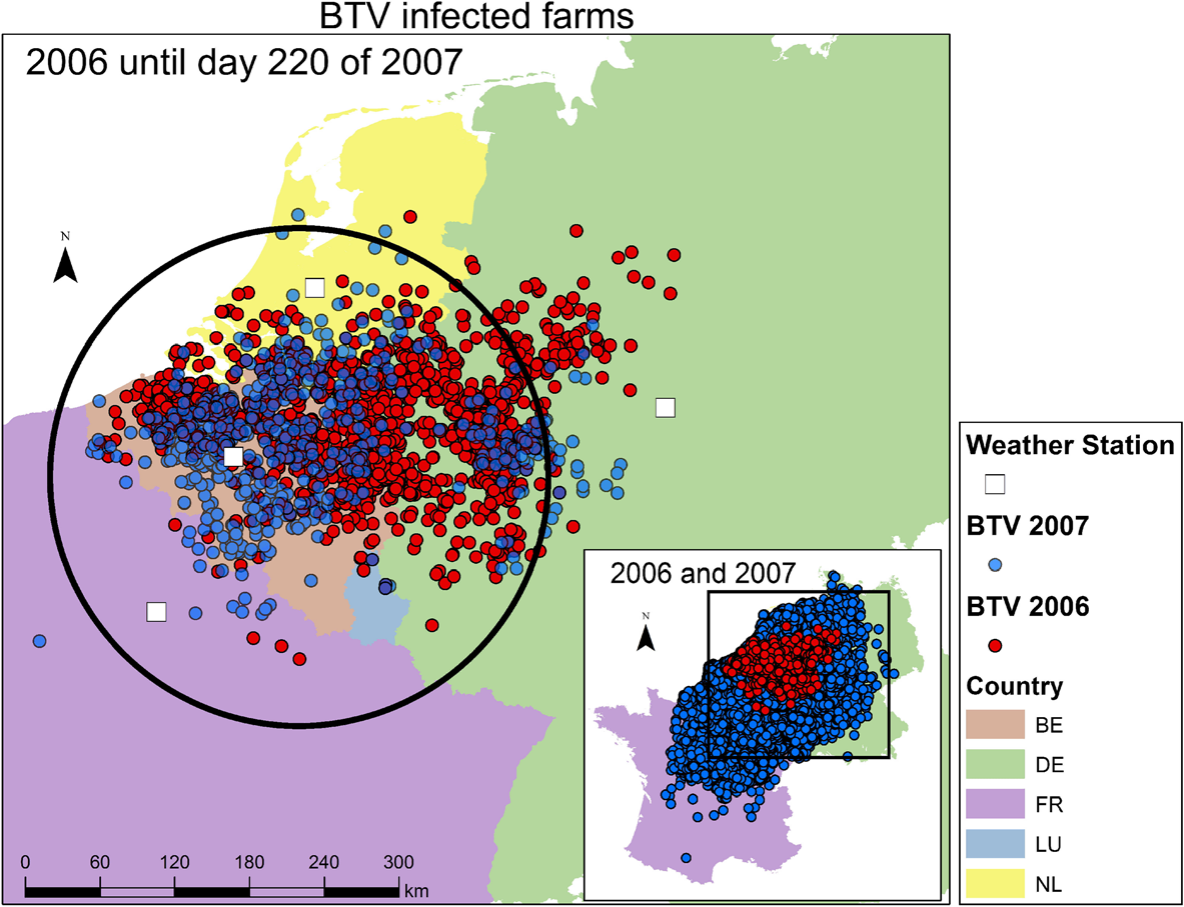
**Map of the 2006 BTV-8 outbreak (Red) and the 2007 outbreak (Blue).** There is a large overlap of the areas containing the cases of the 2006 epidemic (red points) and the cases in the first period (220 days) of the 2007 epidemic (blue points). The black circle is the model approximation of the infected 2006 area. The white squares indicate the locations of the weather stations, from which the temperature data during the epidemic was obtained. In the overview map the spatial distribution of the 2006 (red) and 2007 (blue) outbreak are provided. The 2007 outbreak covers a much larger area than the 2006 outbreak.

The daily mean temperature data during the epidemic period was obtained from four weather stations, with a central location in the affected area per country; De Bilt (Netherlands), Kassel (Germany), Ukkel (Belgium) and Aulnois-SO (France). We restricted ourselves to those four centrally located stations, which are used as temperature reference for the whole country (see Figure 1). The method of analysis does not allow for each farm to be linked to the nearest weather station, since the force of infection links susceptible farms to all farms that are infectious (at the time considered). Thus, the temperature is assessed at country level, without further spatial detail.

### Transmission modelling

The above mentioned data were used to quantify the transmission between herds (1) in terms of a reproduction ratio (only temporal information on the affected herds was used) and (2) in terms of a spatial transmission kernel (both spatial and temporal information required). The first analysis was carried out to evaluate the impact of seasonality and temperature on transmission, while the second analysis aimed at determining the spatial scale of BTV-8 transmission.

A complicating factor in both analyses as compared to the analysis of the 2006 BTV-8 epidemic [2] was that the 2007 epidemic was not a newly arising epidemic, but one advancing from whereto it had already spread in 2006.

### Estimation of reproduction ratio between herds

We estimate the so-called effective reproduction ratio (*R*) between herds, defined as the number of newly infected (herds) initiated by one typical infected (herd), for a given prevalence of immune herds in the population. The effective reproduction ratio equals the basic reproduction ratio multiplied with the susceptible fraction of the population (of herds). In a starting and expanding epidemic, the fraction of susceptibles is close to one, and the effective reproduction ratio is to a good approximation equal to the *basic* reproduction ratio, *R*_*0*_.

The effective reproduction ratio is estimated from the number and chronology of reported case farms, as described by de Koeijer et al. and Bouma et al. [2,10]. In essence, the calculation determines the estimated number of offspring infections for each source farm by determining for each candidate offspring infection the number of possible source herds, and assigning a corresponding proportion of the farm to the source farm in question. We assume that a herd becomes infectious two weeks after introduction of the infection, when the infection has spread substantially in a herd. We assume that the reporting date equals the time at which a herd becomes infectious. From the available data it is not possible to determine in detail when an outbreak farm ceases to be infectious, i.e. when the infection would have died out in both the livestock and surrounding vector population. Since the infection can spread and persist in the livestock and in vectors in and on the farm for several months [11], we therefore assume that all infected farms remain infectious during the whole remainder of the vector-active season.

We analysed the full data set of the 2007 epidemic under these assumptions. To assess if there was a regional effect on transmission we also analysed the data from Belgium, Germany and the Netherlands separately. Furthermore, we tested the impact of the assumption concerning the infectious period by assuming shorter infectious periods.

### Kernel estimation

For the spatial-temporal analysis of the 2006 BTV-8 outbreak [2], we applied the method published by Boender et al. [12,13]. In this method, the inter-farm transmission of a livestock disease is described in terms of a probability as a function of the inter-farm distance. For the formulation of this transmission probability, the transmission kernel is a central component, which is described as a transmission rate *λ*(*r*) across the straight-line inter-farm distance *r*. The estimation of the transmission kernel thus involves calculation of the inter-farm distances between infectious and susceptible farms. In order to calculate these, the location coordinates of non-outbreak farms were set equal to the centre coordinates of their administrative unit. As this approximation to the farm locations was only applied to the non-outbreak farms and not to the outbreak farms, the errors in the calculated distances of susceptible to infectious farms average out, thus having a negligible influence on the estimated distance-dependent transmission risk.

To be as general as possible, while limiting the number of possibilities, we used the following model parameterization for the transmission kernel:

1 $$ \lambda (r)=\frac{\lambda_0}{1+{\left({\scriptscriptstyle \frac{r}{r_0}}\right)}^{\alpha}} $$

in which *r* is the inter-farm distance, α the power, *λ*_0_ the rate of transmission for small distances and *r*_0_ the half-value distance ($\lambda \left({r}_0\right)={\scriptscriptstyle \frac{1}{2}}{\lambda}_0$). The parameterization shown in equation (1) is flexible enough to encompass a range of possible distance dependencies of transmission. In particular, the value of the power α controls whether the transmission is in essence global (α < 2), local (α > 3), or intermediate (2 < α < 3) [2]. In addition, this parameterization has been shown to be preferable to other possibilities, as it produces the best model fit for both the FMD outbreak in the Netherlands in 2001 as well as for the Dutch Avian Influenza epidemic in 2003 [12,14]. In this approach it is assumed that transmission is isotropic (independent of direction), homogeneous (independent of location of the farm) and constant (time independent during the infectious period of the farm). In order to limit the number of estimable model parameters so as to keep the analysis tractable, we make the simplifying approximation that the transmission between farms is farm-size and species independent. Based on these assumptions and on the model parameterization of the transmission kernel, the inter-farm transmission probability can be formulated. The probability of transmission occurring in the infectious period *T* of the source farm to a susceptible farm a distance *r* away equals:

$$ p\left( r, T\right)=1- \exp \left(-\lambda (r) T\right). $$

We used the same assumptions as in the estimation of the reproduction ratio between herds and motivated above, namely that a herd becomes infectious at the time when the first clinical symptoms were observed, that it has a latent period of 14 days before that date, and that all infected farms remain infectious for the whole remainder of the vector-active season. The same assumptions have been employed previously by de Koeijer et al. [2]. The parameters are estimated using Maximum-Likelihood (ML), following the same approach as used in [12]. We calculate 95% confidence intervals for the parameters using the likelihood-ratio test. The likelihood is given by the following expression:

$$ L={\displaystyle \prod_{k\in {\Lambda}^{\mathrm{s}}}}{p}_{\mathrm{esc}, k}\left({t}_{\mathrm{end}}\right){\displaystyle \prod_{m\in {\Lambda}^{\mathrm{i}}}}{p}_{\mathrm{esc}, m}\left({t}_{\inf, m}\right){p}_{\inf, m}\left({t}_{\inf, m}\right). $$

Here the total number of farms is subdivided in two sets: Λ^i^ is the set of all farms that are infected during the epidemic, and Λ^s^ is the set of all farms remaining susceptible. Any farm *m* from the set Λ^i^ escapes from infection until it is infected at time *t*_inf*,m*_ , and any farm *k* from the set Λ^s^ escapes until the end of the vector-active season *t*_end_. The quantity *p*_esc,*m*_(*t*) is the probability that farm *m* is escaping from infection by all infectious farms up to time *t*; which is given by

$$ {p}_{\mathrm{esc}, m}(t)={\displaystyle \prod_{s=1}^{t-1}}{\displaystyle \prod_{j\in {\Lambda}^{\mathrm{i}}}} exp\left(-\lambda \left({r}_{mj}\right){\mathbb{I}}_{\inf, j}(s)\right)\ . $$

Here $\lambda \left({r}_{mj}\right){\mathbb{I}}_{\inf, j}(t)$ is the probability per day that an infectious farm *j* infects a susceptible farm *m* at time *t*, where *r*_*mj*_ is the distance between farms *m* and *j*, and ${\mathbb{I}}_{\inf, j}(t)$ denotes the indicator function which is 1 when farm *j* is infectious at time *t*, and 0 otherwise. The quantity *p*_inf,*m*_(*t*) is the probability that farm *m* is infected by any of the infectious farms at time *t*:

$$ {p}_{\inf, m}(t)=1-{\displaystyle \prod_{j\in {\Lambda}^{\mathrm{i}}}} exp\left(-\lambda \left({r}_{mj}\right){\mathbb{I}}_{\inf, j}(t)\right)\ . $$

We note that the interpretation of the data in terms of infection events is complicated by the fact that the starting point of the epidemic in 2007 was not a population of naïve herds (in which the virus was introduced), but rather a population in which in one area many herds were affected by BTV already in the previous year (2006). About 80% of the farms in Belgium had already been affected by Bluetongue in 2006, while the within-herd prevalence was 24% [15]. Therefore, detected outbreak farms in the 2006 infected area are most probable re-emerging infected, while detected outbreak farms outside this area are most probably susceptible herds becoming infected by means of transmission. In order to assess the importance of local re-emergence, we used the estimation of a spatial transmission kernel as a diagnostic tool. In this analysis (and also in the other analyses performed in this paper) farms are treated as uninfected initially in 2007 even if they were affected before in 2006. If the re-emergence effect dominates, we would expect to find a kernel that shows only a very weak distance-dependence, because the random local re-emergence would in this analysis be interpreted as transmission over random distances. After carrying out this diagnostic procedure we moved on to quantify the between-farm transmission. We correct for the presence of re-emergence by excising the 2006 infected area from the data. To approximate the infected area, a two-step approach was used. In a first step, to study the effect of an approximate excision on the analysis, the infected area was approximated by a single geometric shape. As proxy for the infected area we used the first six months (220 days) of the 2007 epidemic. We approximated the infected area by a circle with a radius of 200 km around the centre of this part of the epidemic. This radius was chosen because 94% of cases was situated inside this circle. All the farms inside this circle were removed from the dataset and the modified dataset was used in the likelihood estimation. The outcome of this first step shows the direction in which the transmission kernel is changing by modifying the dataset. This step justifies the application of a more refined strategy in the next step. In this second step, a more refined approximation of the infected are is used. We assumed that each case farm in 2006 gave rise to an infected area at the start of the 2007 epidemic. Therefore, we removed all farms in a (varying) circle around each farm infected in the 2006 epidemic. With this modified dataset the transmission kernel is re-estimated for excision radii varying from 20 to 160 km.

## Results

### Effective reproduction ratio and temperature influence

**Figure 2:**
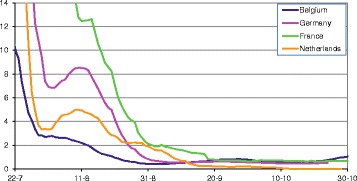
**Estimated reproduction ratio between herds per country in 2007.** Estimate of the reproduction ratio for BTV between herds for different countries, based on the reported case date in 2007, and assuming an infectious period of 2 weeks per herd. In this year there was a secondary warm period in August, which is mirrored by a peak in the reproduction ratio for Germany and the Netherlands. The effect is less pronounced when longer infectious periods are assumed. The y-axis stops at 14. For higher values, the number of infected farms on which the estimate is based, is too small to be considered seriously. Especially in France where the epidemic had hardly started in 2006, the epidemic takes of later than in the other countries and only in September de the case numbers reach a sufficient number.

**Figure 3:**
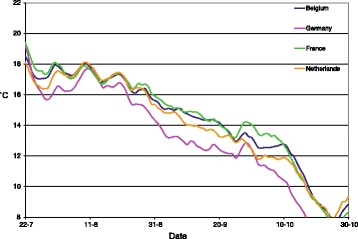
**Average temperature per country.** A 14-days rolling average of the 24 h daily temperatures is shown, to visualise the impact of temperature when comparing with Figure 2. The impact of the declining and subsequently slightly increasing temperature especially in the Netherlands and Germany can be found back in the decline and increase of the reproduction ratio for these countries in August in Figure 2.

The results for the estimated effective reproduction ratio over time are given in Figure 2, separately for the four main affected countries, Belgium, the Netherlands, Germany and France. The date linked to each estimate is the date of reporting, which is assumed equal to the start of the infectious period. In Belgium and France the temperature is quite constant in the first half of august while Germany and the Netherlands experience a secondary warm period around the 10^th^ of August (see Figure 3). This is reflected in the second peak for Germany and the Netherlands in the R graph (Figure 2), which is based on an infectious period of 2 weeks per herd. The effect is less pronounced when longer infectious periods are assumed. Some details of the short-term fluctuations observed in Figure 3 are not mirrored in the results in Figure 2. Whereas the temperature fluctuations in the last week of July and the first week of August do not have a visible counterpart in Figure 2, the more pronounced temperature increase in the second week of August does. Thus we also find here that short-term fluctuations need to be sufficiently pronounced to be detectable with this method.

**Figure 4:**
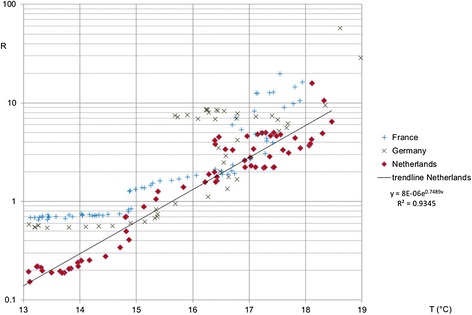
**Correlation between temperature and Reproduction ratio.** The correlation between the reproduction ratio *R* of a herd and the average temperature during the following 14 days *T* for three countries.

In Figure 3, the 14-days rolling average temperature is shown for these countries. Combining the data from these two graphs through the dates, we found that in Germany, the reproduction ratio declines below the threshold, R = 1 (below which one infected farm infects less than one new susceptible farm, thus signifying the decline of the epidemic), on August 30^th^, when the temperature lies around 14.6 °C. In France, this threshold temperature is observed much later, on September 18^th^, when the temperature lies around 14.4 °C. In the Netherlands we find the transmission threshold crossed on September 7^th^ with 14.7 °C. Thus, we confirm the results found from the 2006 epidemic data, i.e. that the temperature needs to be above 15 °C for the virus to spread between herds. Now, comparing the threshold temperature found from the data of the 2006 epidemic, which is approximately equal to 14.8 °C, a strikingly close match to the 2007 results of the first three countries is observed. For Belgium the apparent threshold temperature found for the effective reproduction ratio was substantially higher, on August 21^st^ the threshold was crossed with 17 °C. In Figure 4 we show the correlation between the reproduction ratio of a herd and the average temperature during the following 14 days, in the period July 15^th^ until October 31^st^. In particular the Netherlands shows a strong link (R^2^ = 0.93) between temperature and reproduction ratio. For Germany and France this relationship appears in the higher temperature range, while for the lower temperatures (during fall) this is not the case. We expect that the delayed reporting, which can be expected in newly infected areas, has obscured the relationship. In both countries a substantial area had become newly infected, while in the Netherlands the infection was already present throughout the country. Belgium is not included in Figure 4 because the 2007 outbreaks are mainly due to local re-emergence (see next section), which invalidates the estimated reproduction ratios, as further explained in the Discussion. The temperature threshold which can be read in Figure 4 for the Netherlands at 15.5 °C, is affected by the same effect. Due to a lower susceptibility of the Dutch herds affected in 2006, the reproduction ratio will be underestimated slightly, especially in fall of that year. The threshold for France and Germany, as determined from a trend line on Figure 4 is found around 13 °C, but when all data with average temperatures below 14 °C are removed to correct for the delayed reporting effect, we find the threshold at 14.8 °C for the French data and at 14.7 °C for Germany (trend lines not shown in Figure 4).

### Spatial transmission kernel

**Figure 5:**
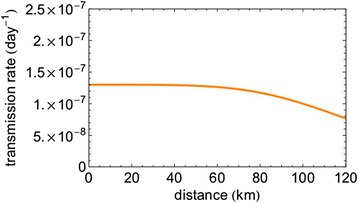
**Transmission kernel obtained from full 2007 data.** This kernel shows almost distance-independent “transmission” over a large spatial range (up until about 80–100 km).

As can be seen from Figure 1, there is a large overlap of the areas of the 2006 epidemic and the cases in first period of 2007. With that, many of the new cases in the first period of 2007 may result not from between-farm transmission in 2007, but instead from persistence and subsequent local re-emergence of the infection in the area already infected in 2006. As explained in the Materials and methods, the estimation of a spatial transmission kernel can serve as a diagnostic tool to assess the importance of such local re-emergence. A joint analysis of the complete dataset of 2007 as one epidemic results in the kernel that is presented in Figure 5, and shows almost distance-independent transmission over a large spatial range (up until about 80–100 km). The estimated parameter values are *λ*_0_ = 0.13 (10^−6^ day^−1^), *r*_0_ = 130 (km) and *α* = 4.6. This corresponds to (almost) random “transmission” over space, which is rather implausible. Therefore, the assumption that the all outbreak farms are caused by transmission in 2007 must be invalid. Thus, this kernel estimation functions as a diagnostic tool for the 2007 outbreak, indicating that the pattern analysed is dominated by outbreaks due to local re-emergence rather than by outbreaks due to actual between-farm transmission.

**Figure 6:**
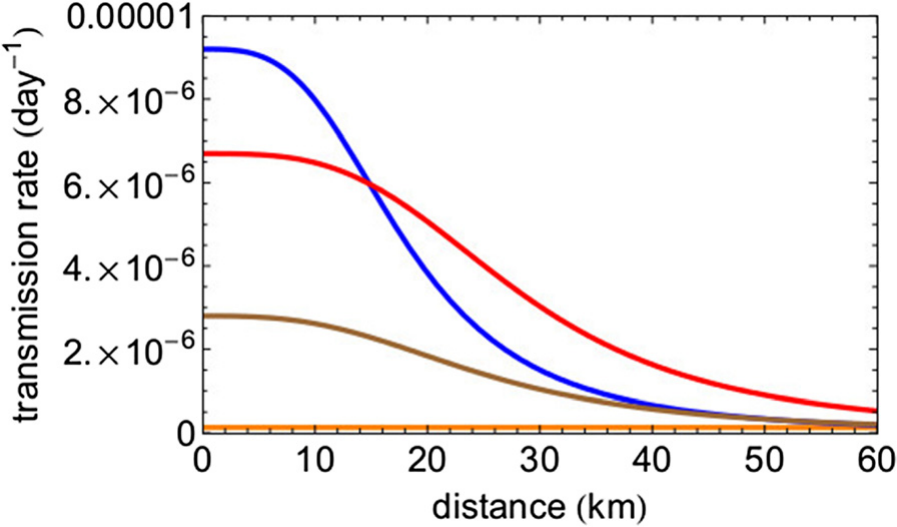
**Spatial transmission kernel estimated for BTV-8 datasets.** The kernels are estimated for the unmodified 2007 dataset (orange), the dataset in which the 2006 outbreak area is removed by taking out a circle of 200 km radius (brown) and the dataset in which the 2006 outbreak area is removed by taking out circles of 80 km radius around each case of the 2006 outbreak (red). The 2006 German kernel is added for comparison (blue).

**Figure 7:**
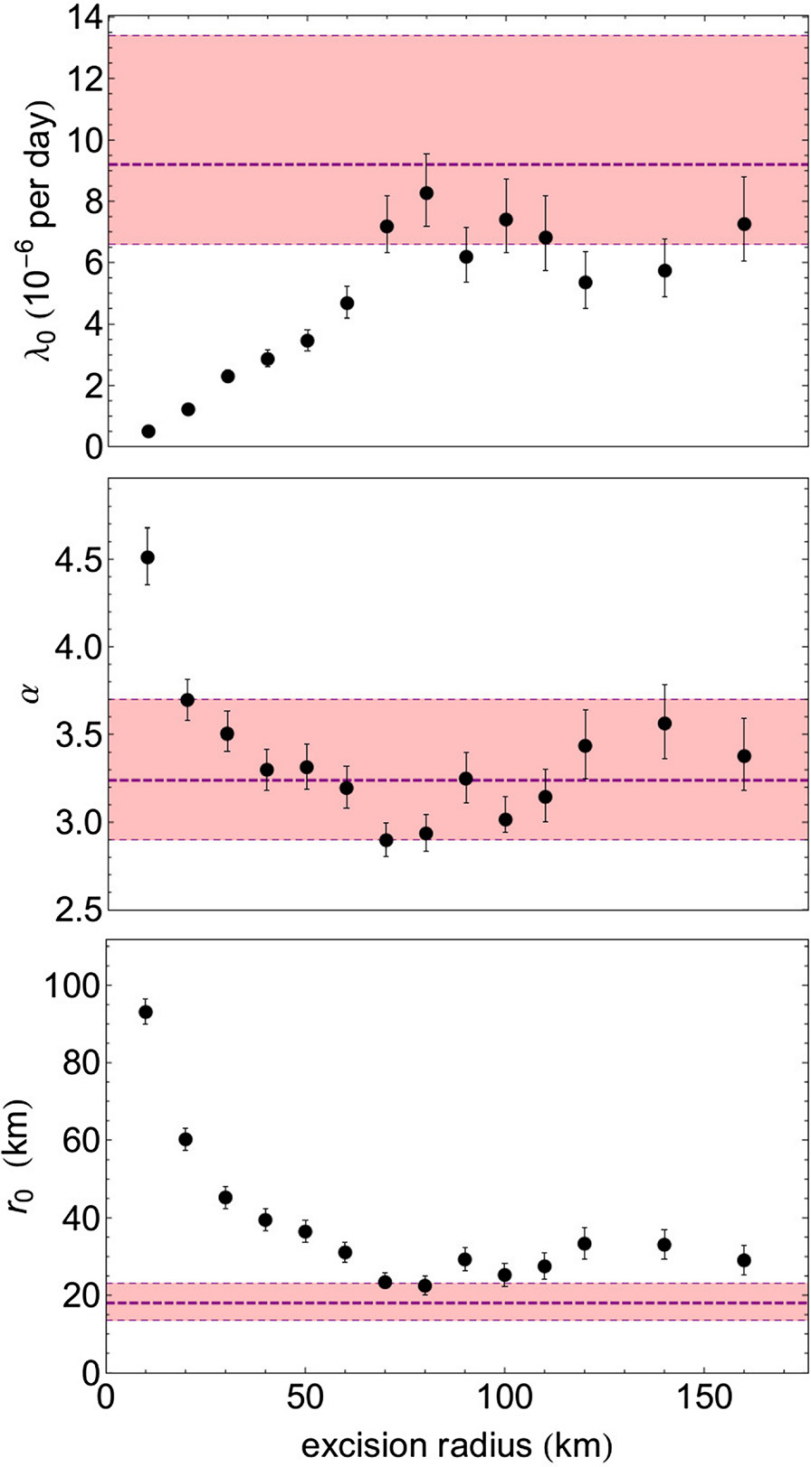
**Maximum Likelihood (ML)-estimates for the transmission rate parameters for the modified 2007 dataset.** The transmission rate parameters (*λ*_0_, *α* and *r*_0_) during the BTV-8 epidemic in 2007 are estimated for the modified 2007 dataset, in which the 2006 outbreak area is removed by taking out circles with varying excision radius around each case of the 2006 outbreak. The parameter value for the 2006 German kernel is added for comparison (dashed line).

We tried to separate the two types of transmission by eliminating the potential re-emergence area from the dataset, using the two-step approach described in the Materials and Methods section. First, we excluded this area by means of removing from the data all the farms within a circle with a radius of 200 km around the centre of the 2006 epidemic (see Figure 1). This results in a transmission kernel that does show distance dependence. This distance dependence is quite similar to that seen in the transmission kernel derived for Germany based on the 2006 data (see Figure 6). In particular the large-distance behaviour, as governed by the parameter *α*, is similar to that for the Germany-2006 data (although not the same, see Table 1). Second, to make the approximation more precise we proceeded further along the same line by removing all farms in a circle around each farm infected in the 2006 epidemic. Subsequently we estimated the kernel parameters for various radii of the circles. The results are given in Figure 7, where we can see that the kernel parameters values level off for excision radii larger than 80 km. This implies that the excision radius should be at least 80 km to remove all reemergence areas. For larger radii also new transmission events are removed from the calculation, leaving the point estimate for the estimated kernel parameters unchanged. The minimum excision radius required provides evidence that an infected farm in 2006 in fact represents an infected area with a maximum radius of 80 km. Above this radius, the estimated kernel parameters, in particular the parameter *α*, stabilize as a function of the radius, suggesting that only above this radius the area of re-emergence has been fully removed. The parameter *α* stabilizes at a value similar to the kernel parameter value of the German 2006 epidemic. An overview of the parameters of the different kernel estimations is presented in Table 1.

**Table 1:** **Maximum Likelihood (ML)-estimates for the transmission rate parameters during the BTV-8 epidemic in 2007**

**Dataset**	***λ***_**0**_ **(10** ^**−6**^ **day** ^**−1**^ **)**	***r***_**0**_ **(km)**	***α***
Germany 2006	9.2 (6.6-13.4)	18.0 (13.5-23.0)	3.2 (2.9-3.7)
Europe 2007 without 2006 infected area	3.2 (2.9-3.8)	21.8 (19.5-24.3)	2.6 (2.57-2.62)
Europe 2007 without 2006 infected farm areas	8.3 (7.2-9.5)	22.5 (20.2-24.9)	2.9 (2.8-3.0)

Parameter values estimated from the 2007 dataset in which the 2006 epidemic area is removed by taking out an area with a radius of 200 km, and from the 2007 dataset in which the 2006 epidemic area is removed by taking out areas with a radius of 80 km around each case of the 2006 epidemic; the parameter values estimated for the 2006 German kernel are added for comparison.

## Discussion

The temperature thresholds estimated from the 2007 data for Germany, France and the Netherlands was found to agree within 0.5 °C with the value of approximately 14.7 °C that was estimated for 2006. This suggests that the temperature effect is very influential, and that variation in for example the density of *Culicoides* between years has less influence, despite its strong impact on the reproduction ratio of the infection [16]. Since we found the same results in various countries and in two different years with a very different temperature profile (2006 featured a heat wave in July) [17], we can conclude that this transmission under field conditions is strongly dependent on the temperature conditions, at least within NW Europe. In this part of the analysis we focussed on the temperature effect on transmission, since this is known to have a major impact [2]. In doing so, we ignored the impact of other risk factors, such as the related impact of elevation [18] to obtain the most straightforward results on temperature.

The results nicely compare to conclusions from Carpenter et al. [19], who find that a minimum temperature of about 13 °C is required for replication of BTV strains in *Culicoides,* depending on the specific viral strain and vector species. Obviously a higher temperature is required if the replication is to be sufficient for an expanding epidemic, while minimal replication (around 13 °C) will only lead to occasional transmission without epidemic behaviour and mostly restricted within herds. Gubbins et al. [20] suggests a within-herd temperature threshold around 15 °C and de Koeijer and Elbers [16] estimate a higher temperature (but with high uncertainty). For transmission between herds we expect a higher temperature threshold than for transmission between animals, because major epidemics within the herds are a requirement for (observing) a major epidemic between herds in such mainly locally spreading infections. Our present results thus suggest that these two older publications somewhat overestimated the relevant temperature. However, given the limited input information (only literature before the NW-European epidemic was used), especially the estimate by Gubbins et al. was very close. Transmission between herds is also expected to be less sensitive to the vector-host ratio, because it takes place over a longer time period and a larger spatial scale, explaining better the consistency between these findings and the earlier results of de Koeijer et al. [2]. Regarding the risks for re-emergence of this virus in Europe, we need to take this threshold into account. Our results can be used to provide input for predictive models on temperature and climate effects, such as described in a review by Baylis who explains the major influence of climate on vector-borne infections in general and BTV specifically [21]. Guis et al. show which areas in Europe are most at risk for re-emergence of BTV, and also make some forward projections, indicating that Europe needs to remain on guard for this and similarly spreading infections [3].

While several authors [2,22] reported temperature as an important risk factor for the 2006 BTV epidemic in Belgium, this had not been reported as such for the 2007 epidemic. Naïve calculation using the methodology explained in this paper produces a far higher temperature threshold in Belgium than in the other three countries. However, as the spatial analysis has indicated, the new cases in Belgium in 2007 arise mostly from local re-emergence rather than from between-herd transmission. For that reason the calculation of the effective reproduction ratio becomes invalid, and thus we cannot derive any conclusions here concerning the threshold temperature for Belgium. Other methods are more suitable for answering such questions [3,22]. From the overall 2006 dataset, the actual spatial transmission was elucidated in [2] only after taking into account the spatial heterogeneity in control measures. For the 2007 epidemic analysed here this was not an issue, as the control strategies in the newly infected areas were more or less comparable and in line with the EU regulations. However, in the analysis of the 2007 data it was necessary to correct for widespread virus re-emergence. To explain the overwintering of BTV, several transmission routes have been proposed, e.g. by means of infected adult vectors, latent infected cattle, transplacental infection, but the mechanism involved for overwintering is still poorly understood [23]. Our results indicate that in 2007 the epidemic did not emerge at one location, but re-emerged after the winter throughout the area affected during 2006, an area in which the population had already been infected to a large extent.

In contrast, for the 2006 epidemic, it is most likely that the epidemic emerged at one location, from which it spread into a wholly susceptible population. The different countries implemented different control measures and this led to different spatial kernel for the different countries [2].

In the transmission kernel estimation we did not take into account the temperature dependence, nor did we attempt to quantify farm-size and/or species dependences of the kernel. By assuming a constant kernel parameter λ_0_ we model the average kernel across the transmission season and across farm sizes and species. The reason for this simplification is that incorporation of the dependencies comes with the cost of introducing additional estimable parameters, increasing computational intensity of the parameter estimation. This cost was deemed too large as we were mainly interested in analysing the distance dependence of transmission risk. In this context, it is interesting to note that in a kernel estimation for classical swine fever transmission between pig farms, it was found by Boender et al. [24] that incorporation of a farm-size dependence within the kernel leads to a better fit, but does not change the estimated distance dependence of the transmission risk.

The fact that the shape of the transmission kernel found for 2007 is very similar to that found for Germany-2006, despite the different year and the different geographical areas affected, supports the hypothesis that the shape of the transmission kernel is mainly determined by the type of control measures applied. This is because we consider the large-distance behaviour of the transmission kernel for Germany-2006 as representative of transmission under EU control measures in 2006. The rationale for this is that by de Koeijer et al. [2] it was found that the shape of the kernels estimated for Belgium before 24 August 2006 (lift of the movement restrictions) and that for Germany-2006 are very similar, whereas the kernel for Belgium after 24 August 2006 is completely different. For completeness we note that de Koeijer et al. [2] the kernel for The Netherlands in 2006 was found to be different from Germany-2006 and Belgium-before-24/08/2006, although movement restrictions were applied in The Netherlands throughout the year. Our hypothesis is that this is caused by spill-over effects from the transmission wave taking place from east to west in Belgium after 24 August 2006, as visualized by Ensoy et al. [25]. Our results suggest that to fully remove the area of re-emergence one has to excise circles with a radius of about 80 km around all farms that were already infected in 2006. In view of this, the question arises what type of unit actually is the natural epidemiological unit within the population from the point of view of BTV-8 transmission. Our results suggest that the unit is not a farm, but is an area of considerable size around the farm. Should a non-vaccination strategy be considered for BTV-8 in future, then this implication would be important input for the assessment of freedom from infection and for the development of a supporting control program. To prove freedom from infection not only the detected farms should be regarded, but also the animal population in the area around these farms should be sampled. Further research into the distribution of infection in infected areas may help in determining optimal control measures in future.

In summary, our findings are as follows. The temperature threshold and the spatial transmission kernel obtained by de Koeijer et al. [2] are found to be of more general significance, i.e. beyond 2006 and beyond the area affected in 2006. Furthermore, we have shown that within the area affected already by the 2006 epidemic, the pattern of newly infected farms in 2007 is due mainly to local re-emergence of the virus, indicating that persistence through winter was ubiquitous for BTV-8. Methodologically these analyses for 2007 were more challenging than the earlier ones for 2006, because of the need to take into account that the 2007 epidemic was advancing from a large area across which it had already spread in 2006.
